# Abnormal 18F-FDG Uptake Detected with Positron Emission Tomography in a Patient with Breast Cancer: A Case of Sarcoidosis and Review of the Literature

**DOI:** 10.1155/2009/785047

**Published:** 2009-10-01

**Authors:** Selmin Ataergin, Nuri Arslan, Ahmet Ozet, Mehmet Ali Ozguven

**Affiliations:** ^1^Department of Medical Oncology, GATA (Gulhane) Faculty of Medicine, Etlik, 06018 Ankara, Turkey; ^2^Department of Nuclear Medicine, GATA (Gulhane) Faculty of Medicine, Etlik, 06018 Ankara, Turkey

## Abstract

18F-FDG PET is a useful and sensitive imaging method for a variety of malignancies, however, the specificity is low in active infections and inflammatory diseases. We describe a female patient with stage IIIA breast cancer in first complete remission with combination chemotherapy who developed nodular formations in the lung and axilla 12 years later. Imaging studies as well as FDG PET showed nodular lesions and increased metabolic activity which was interpreted as the progression of the primary disease. She was first given combination chemotherapy and hormonal therapy but was proven thereafter to have sarcoidosis by pathologic examination and was successfully treated with corticosteroid treatment.

## 1. Introduction

Sarcoidosis is a chronic multisystem disorder which is characterized by the accumulation of T lymphocytes and mononuclear phagocytes, noncaseating epitheloid granulomas in affected organs, and derangements of the normal tissue architecture. Although the etiology is unknown, it is thought to be caused by exaggerated cellular immune responses. The first pathologic manifestation of the disease is the accumulation of mononuclear inflammatory cells, mainly CD4^+^ T helper 1 lymphocytes and mononuclear phagocytes in the affected organs. This inflammatory process is followed by the formation of granulomas, aggregates of macrophages, and epitheloid cells, as well as multinucleated giant cells [1]. There is no established relation between sarcoidosis and cancer, however, they can coexist. Battesti described seven cases of cancer in 580 patients with sarcoidosis [2]. Several types of tumors have been reported to be involved [3]. The observation of sarcoidosis after cancer would suggest a relation between both conditions.

Differentiation of malignant from benign nodules is a common dilemma in diagnostic imaging. Metabolic imaging with 2-(18F)-fluoro-2-deoxy-D-glucose (FGD) positron emission tomography (PET) is being used more and more to differentiate benign from malignant focal lesions [2, 3] and it has been shown to be more efficacious than conventional computed tomography (CT) [4]. However, FDG is not a cancer-specific agent, and benign diseases related mainly to infection or inflammation also can show false-positive intense FDG uptake which causes difficulty in differentiating benign disorders from malignant diseases [3–7].

## 2. Case Report

**Figure 1:**
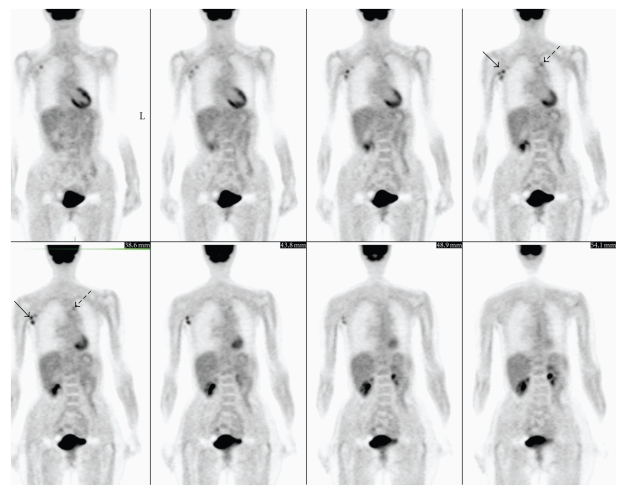
Coronal FDG-PET images showed a focus of mildly increased FDG activity (SUV max = 2, 1) in the medial segment of the upper lobe in the left lung (dotted arrows) and multiple moderate to markedly increased FDG foci (SUV max = 3, 4) in the right-sided axillary lymph nodes (arrows) in a 63-year-old woman, who underwent FDG-PET scan for the evaluation of enlarged right axillary lymph nodes and lung nodules, for the detection of recurrent metastatic breast cancer disease. Histopathologic examination of the axillary and endobronchial lesions revealed sarcoidosis.

A 63-year-old woman was diagnosed T3N1M0 right-sided breast cancer 12 years ago and achieved a complete remission after the administration of 6 courses of CAF (cyclophosphamide, doxorubicin, 5-FU) chemotherapy and radiotherapy. On her admittance to another institution twelve years later, right-sided minimal pleural effusion was detected on CT. Although cytologic examination of the pleural fluid was negative for malignancy, CA-15-3 level was slightly elevated and CEA level was within the normal ranges. Any pathogen could not be detected by microbiological examinations. Due to a previous history of breast cancer, a chemotherapy regimen consisting of paclitaxel and farmarubicin was initiated. Pulmonary nodular lesions measuring 1 cm in the left lung, and axillary lymph nodes of 0.8 cm were detected on control CT after 4 months, but the patient refused a biopsy. Letrozole was initiated during the persuation period of the patient for biopsy. F-18 FDG PET revealed abnormal FDG accumulation in the left lung with mild metabolic activity (SUV max: 2.1), and also moderately high metabolic activity (SUV max: 3.4) related to multiple lymph nodes in the right axilla (Figure 1). Finally, the patient was convinced for an excisional biopsy from axillary lymph node as well as endobronchial lymph node biopsy, which were revealed sarcoidosis. All pathologic imaging findings regressed after corticosteroid treatment.

## 3. Discussion

We described a female patient with stage IIIA breast cancer who developed nodular formations in the left lung and in the right-sided axillary lymph nodes 12 years after the achievement of a complete response. FDG PET showed low to moderately increased metabolic activity which was first interpreted as the progression of the primary disease, but confirmed thereafter pathologically to be sarcoidosis.

Metabolic imaging with FDG-PET has gained an important role in the management of oncologic diseases. However, false positive FDG uptake or false-negative PET scans are frequently encountered. Proper interpretation and accurate characterization of an abnormality can be accomplished only if one is aware of possible false-positive or negative conditions. FDG PET is able to image the metabolic differences between normal and malignant cells according to glucose metabolism of a lesion, which can be helpful in differentiating benign and malignant lesions [14]. Malignant cells demonstrate higher glucose metabolic activity than benign lesions do. Moreover, the total of FDG uptake is not completely within the tumor cells themselves. The newly formed granulation tissue around the tumor and the macrophages infiltrating heavily at the marginal areas surrounding the necrotic area of the tumor may show high FDG uptake. On the other hand, active granulomatous processes such as tuberculosis [3, 5, 6], fungal infections [7], and sarcoidosis [8–13] have been reported to accumulate FDG and cause false-positive PET scans for malignancy (Table 1). High FDG uptake in activated inflammatory cells is due to markedly increased glycolysis and the hexose monophosphate shunt which is stimulated by phagocytosis, with increases of 20–30 times of baseline values [15]. As a result, approximately 24% of the FDG concentration in a tumor mass is derived from nontumor tissue [16]. Therefore, acute or chronic infection or inflammation must always be considered as a potential false-positive finding when whole-body F-18 FDG PET scans are interpreted, especially in patients with previous history of cancer.

In sarcoidosis, the cellular infiltrate is composed of lymphocytes, macrophages, and epitheliod cells, and can involve almost any organ. The disease process is believed to initially involve pulmonary interstitial tissue with the formation of noncaseating granulomas, which are characteristic of the disease. In sites of active inflammatory disease, rapidly dividing cells, such as inflammatory cells, have a high glycolytic activity. Besides, macrophages are known to have high rates of protein secretion and membrane recycling, resulting in increased FDG uptake. As expected, the role of FDG PET imaging in the diagnosis of sarcoidosis is limited. However, in a patient with proven sarcoidosis, both the extent of involvement and disease activity can be more accurately assessed by FDG PET than other methods such as Ga-67 Sitrate scintigraphy [17]. 

As discussed earlier, pulmonary granulomatous diseases, such as tuberculosis, sarcoidosis, fungal infections, and also any kind of infection, may predispose to localized FDG thoracic uptake, and limit the specificity of FDG PET scan by mimicking metastases. Although being nonspecific, given the patients young age, and lack of history of primary malignancy, the findings of multiple bilateral pulmonary nodules with associated mediastinal and symmetrical hilar lymphadenopathy on FDG PET scan are more suggestive of a granulomatous disorder, or an infectious process rather than pulmonary metastases. In a patient on a complete remission after treatment for low-grade tumor pathology, similar FDG PET findings are more suggestive for benign conditions, especially if the patient has normal clinical examination with no other radiologic or laboratory abnormality indicating recurrence. However, as in this particular case, since both sarcoidosis and breast cancer may have the potential to invade the lymphoid systems throughout the body, the uptake pattern on FDG PET images may be nonspecific, and cannot differentiate granulomatous disease from malignant conditions.

Many benign conditions that can cause high uptake of 18-FDG and have the potential for false-positive interpretation in oncologic studies have previously been described [18]. These false-positive FDG uptakes may mimic specific cancer types according to its localization. For example, focal asymmetric neck muscle uptake, focal bowel uptake, or focal ureter uptake may mimic cervical, mesenteric, or peritoneal and paraaortic or iliac lymph node metastases, respectively. Moreover, lymphatic drainage of the extravasated tracer from the injection site to the axillary lymph nodes may cause misinterpretation of the FDG PET scans as axillary lymph node metastasis. In the skeleton, diffuse increase in bone marrow activity seen as a result of chemotherapy or colony stimulating factors is now well recognized. Thymus hyperplasia may also mimic thymic disease and anterior mediastinal involvement. On the other hand, differential diagnosis of normal ovarian and endometrial uptake from gynecological benign or malignant disease is a well-known problem. In many instances, these physiologic variants and benign pathological causes of FDG uptake can be specifically recognized and properly categorized; in other instances FDG uptake may reflect nonspecific clinical importance [18, 19].

Conversely, there are a number of malignant tumors that show little or no FDG uptake decreasing the negative predictive value of FDG PET scan. For example, very small lung metastases may be beyond the resolution of current PET scanners. Mucosa-associated lymphoid tissue lymphomas, small lymphocytic cell lymphoma, and well-differentiated neuroendocrine tumors may show only low-grade activity that may be inconspicuous. On the other hand, sclerotic bone metastases, renal cell carcinoma, and brain metastases also can be inconspicuous on occasion, either because of low uptake or high surrounding background activity [19].

The most widely applied PET radiopharmaceutical in oncology is ^18^F-FDG. However, ^18^F-FDG PET is not 100% accurate in the detection of primary tumors and their metastases. Therefore, more specific PET radiopharmaceuticals are needed. To increase the accuracy of FDG PET imaging, several PET tracers other than 18F-FDG have been studied. The fact that amino acid imaging is less influenced by inflammation may be advantageous in comparison with ^18^F-FDG PET imaging, although tumor specificity is not absolute [20]. Among the tracers other than ^18^F-FDG, C-11-labelled amino acid agents are very promising. Amino acid transport is generally increased in malignant cells and amino acid imaging is less influenced by inflammation. For example, C-11 thymidine is used in the evaluation of tumor DNA replication and cell division rates, and C-11 choline (^11^C-CHOL) is an agent which reflects cell membrane synthesis. These agents may contribute for the differential diagnosis of FDG uptake in patients suspicious for malignancy [20, 21].

The presented case should be an educational case in terms of demonstrating the importance of patient-doctor collaboration, and all patients should be convinced for a biopsy in suspected situations before the initiation of a definitive anticancer treatment. In conclusion, if any abnormal FDG accumulation is detected on FDG PET study in lymph nodes or parenchyma of a patient with a previous history of cancer, the diagnosis of cancer should always be confirmed pathologically to exclude false-positive FDG PET findings. 

## Figures and Tables

**Table 1:** Published sarcoidosis cases detected by 18 F FDG-PET* study in cancer patients.

Reference	Author	Publication year	Primary cancer	Number of cases	Age	Sex
[8]	Takanami et al.	2008	Esophagus cancer	2	48 and 50	M
[9]	Li et al.	2007	Lymphoma	1	45	F
[10]	Kunstman et al.	2007	Ovarian cancer	1	55	F
[11]	Maeda et al.	2005	Lung cancer	1	66	M
[12]	de Hemricourt et al.	2003	Hodgkin's disease	2	38	M
[13]	Muggia et al.	1998	Seminoma	1	41	M

*2-(18F)-fluoro-2-deoxy-D-glucose positron emission tomography.
